# Lipid Status of the Two High Latitude Fish Species, *Leptoclinus maculatus* and *Lumpenus fabricii*

**DOI:** 10.3390/ijms14047048

**Published:** 2013-03-27

**Authors:** Svetlana A. Murzina, Zinaida A. Nefedova, Stig Falk-Petersen, Pauli O. Ripatti, Tatiana R. Ruokolainen, Svetlana N. Pekkoeva, Nina N. Nemova

**Affiliations:** 1 Environmental Biochemistry Lab, Institute of Biology, Karelian Research Centre of the Russian Academy of Sciences, Pushkinskaya st., 11, Petrozavodsk 185910, Russia; E-Mails: znefed@krc.karelia.ru (Z.A.N.); ripatti@bio.krc.karelia.ru (P.O.R.); truok@krc.karelia.ru (T.R.R.); nemova@krc.karelia.ru (N.N.N.); 2 Akvaplan-niva, Fram Centre, Hjalmar Johansens gt. 14, Tromsø NO-9296, Norway; E-Mail: stig@npolar.no; 3 Faculty of Biosciences, Fisheries and Economics, University of Tromsø, Tromsø NO-9037, Norway; 4 Petrozavodsk State University, Lenina st., 33, Petrozavodsk 185910, Russia; E-Mail: pek-svetlana@mail.ru

**Keywords:** biochemical adaptation, lipids, phospholipids, fatty acids, fishes, Stichaeidae, Arctic, sub-Arctic

## Abstract

A comparative study of the lipid status (*i.e.*, the total lipid and phospholipid concentrations and the percentage of fatty acids of the total lipids) of adult specimens of daubed shanny (*Leptoclinus maculatus*) from Svalbard waters (Isfjord) and slender eel blenny (*Lumpenus fabricii*) from the White Sea (Onega Bay and Tersky shore) was performed to study the metabolism and functions of lipids of these fishes in ontogeny and under various ecological conditions. Slender eel blenny from both areas of the White Sea were distinguished by a high level of sphingomyelin compared with the daubed shanny from Svalbard, and the amount of total phospholipids was higher in slender eel blenny from Onega Bay than in slender eel blenny from the Tersky shore. The extent of saturation and the signature of polyenic fatty acids varied according to the specific species of the Stichaeidae family under study. These results demonstrate the differences in the trophoecological and hydrobiological conditions of habitations of these species and highlighted the importance of considering certain trends in the lipid profiles of these fishes as specific features of the organization of the ecological and biochemical mechanisms of adaptation.

## 1. Introduction

One fundamental challenge in biology research is the study of the mechanisms of adaptations of an organism to its environment. All adaptive processes are based on biochemical adaptation, *i.e.*, the ability of living systems to adapt to changing environmental conditions through modifications of their biochemical structures and metabolic reactions. In addition, the integrity and functional activity of macromolecules and supramolecular complexes should be maintained during this process [1,2]. Lipids and their components play an important role in the biochemical adaptations of living organisms that dwell in the severe and unique conditions of the northern latitudes. These lipids are of major importance in the ecological and biochemical monitoring and testing of aquatic organisms. The role of lipids in cellular metabolism is versatile, although three main functions have been identified: energetic, structural and bioeffector roles (*i.e.*, lipids acting as messengers). The differences between the lipids of marine organisms that inhabit the high Arctic compared of those of moderate latitudes are quantitative rather than qualitative. A change in the lipid structure is one aspect of the adaptive reactions that provide an organism with the ability to survive under the conditions that result from various combinations of ecological factors (within physiological limits). A compensatory lipid mechanism allows for the maintenance of membranes (e.g., fluidity, permeability, mobility of membrane components, activity of ionic transport and activity of membrane enzymes) such that the membranes are able to optimally fulfill their diverse functions [2–4]. To understand how organisms are able to survive in constantly changing environments, it is necessary to determine the roles of individual metabolites and lipids in particular in the physiological and biochemical features of hydrobionts.

Daubed shanny (*Leptoclinus maculatus*) and slender eel blenny (*Lumpenus fabricii*) are fishes of the Stichaeidae family that are widely distributed in Arctic and sub-Arctic marine ecosystems. Only a small number of studies on the biology, morphology and ecology of certain members of this family have been previously published [5–11]. The larvae of daubed shanny are pelagic the first years and later gradually move to benthic environments during their development into adult fish [10]. During its change from a pelagic to a bottom environment, the daubed shanny faces changes in its environment (temperature, light, salinity, currents and pressure), food sources and type of feeding. *Leptoclinus maculatus* and *Lumpenus fabricii* are important in high latitude food webs, in which these species (especially their fatty larvae) are major components of the diet of marine predators, including commercial fishes, mammals (seals) and birds [12–14]. The fishes studied in this investigation have a dual niche in Arctic trophic webs as both prey and predator [15]. The available data on the lipid and fatty acid profiles and the dynamics of the lifecycle of daubed shanny remain rare [7,11]; in addition, data on slender eel blenny are not available.

Comparative research on lipid and fatty acid profiles and lifecycle dynamics may be important not only for understanding the fundamental basis of biochemical adaptations of marine organisms to life in the North, but also for identifying metrics for monitoring the status of high-latitude ecosystems that experience changing environmental factors.

A comparative study of the lipid status, which is based on the total lipid content, including phospholipids, the fatty acids components of the total lipids and the lipid classes, in adult specimens of *Leptoclinus maculatus* from the shallow waters of northwestern Svalbard (Isfjord; high-Arctic) and *Lumpenus fabricii* from the White Sea (Onega Bay and Tersky Shore; sub-Arctic) was performed. The results reveal differences in the total lipid levels. The lipid classes and fatty acids that were determined in the fish species from the high-Arctic and sub-Arctic regions allow for the identification of these lipids as specific features of the ecological and biochemical mechanisms of adaptation of these organisms.

## 2. Results and Discussion

The total lipid (TL) levels did not vary significantly in the fishes from high latitudes and ranged from 11.4% to 11.8% dry weight (dw) (Table 1). It is likely that the environmental conditions of both ecosystems (high-Arctic and sub-Arctic) in the autumn are similar and do not play a major role on the TL content in the studied fishes. This lack of a difference in the TL content may also indicate that the TL levels are genetically determined.

Total lipid level in the flesh of fishes under study did not vary significantly, while the composition of lipid classes changed. Among lipid classes, triacylglycerides (TAGs) were abundant (*p* ≤ 0.05) in the slender eel blenny from Tersky Shore of the White Sea (5.8% dw), which can be explained by the distinguished environment (food in particular) compared to other sampling areas. It was assumed that *Leptoclinus maculatus* as a winter spawner depending on lipid reserves can prolong the spawning period until spring [11]. So, we suggest that muscles store lipids mostly for their own needs and there is less supply of them for gonads or liver, which are connected to each other in terms of reproduction [16]. The high level of cholesterol (CH) (3.6% dw) and phospholipid (PL) in *Leptoclinus maculatus* from Svalbard showed modifications in the microviscosity of membranes caused by temperature (Table 1). A higher level of PLs (corresponding to the level of PI, phosphatidylserine (PS) and lysophosphatidylcholine (LPC)) was detected in the sub-Arctic *Lumpenus fabricii* from Onega Bay of the White Sea (6.8% dw) compared to that of the Arctic *Leptoclinus maculatus* and the sub-Arctic *Lumpenus fabricii* (Table 1) from the Tersky shore of the White Sea (4.7% and 4.4% dw, respectively). The relative concentrations of the most abundant PLs (phosphatidylcholine (PC) and phosphatidylethanolamine (PEA)) were higher in the high-Arctic daubed shanny and the sub-Arctic slender eel blenny from Onega Bay than in the sub-Arctic slender blenny from the Tersky shore of the White Sea. The concentration of sphingomyelin (SFM) and the PC/PEA ratio in *Lumpenus fabricii* from both areas of the White Sea were equal and much higher relative to those of the *Leptoclinus maculatus* from Isfjord (Svalbard) (Table 1). The detected differences in the SFM contents in the studied species from the Stichaeidae family that dwell in sub-Arctic and high-Arctic environments might be explained by inheritable factors. It is thought that SFM first arose in free-living worms [17], and the level of this PL in marine organisms is known to be associated with the extent of evolutionary development. The relatively low SFM concentrations and PC/PEA ratios in the Arctic daubed shanny can be considered a specific feature of the ecological and biochemical mechanisms of adaptation that alter the metabolism of membrane lipid levels at low temperatures (0 °C) (Table 1).

It is known that the PC/PEA ratio of an organism decreases during thermal adaptation and acclimation. In addition, the extent of unsaturation of PEA increases, due to its fatty acid constituents; as a result, the physico-chemical properties and the structure of the biomembrane change to maintain the optimal conditions for the activities of the membrane-connected enzymes [2,18,19]. The quantitative variations of PLs (mainly PEA, phosphatidylserine (PS) and PI) and their fatty acids provide the required membrane fluidity, and many processes of adaptation to changing temperature are associated with these variations [4,20,21].

The role of individual PLs in the adaptation of cold-body organisms involves many different regulatory functions and mechanisms. PLs ensure the activity of numerous membrane-bound enzymes. One of the mechanisms by which the activity of an enzyme can be altered is the modification of the surrounding lipids, which often have specific functions [3,22–24].

The higher level of PLs (corresponding to the PI, PS and LPC contents) in *Lumpenus fabricii* from Onega bay of the White Sea compared with that of *Lumpenus fabricii* from the Tersky shore and *Leptoclinus maculatus* from Isfjord may be explained by changes in the microviscosity and ion permeability of biomembranes, resulting from different environmental conditions (temperature, salinity and water pressure). It is known that PI and its metabolites are mediators and messengers in diverse essential signaling pathways, such as intracellular Ca^2+^ turnover and general adaptation to ecological factors [2,25]. An increase in the concentration of PS, which is an unsaturated PL, induces the activity of membrane-associated enzymes, such as Na^+^- and K^+^-ATPase, which are important in osmoregulation [2,26]. It was reported [27] that there are differences in the level of certain PLs (particularly PI) and fatty acids in the larvae and adults of Arctic daubed shanny *Leptoclinus maculatus* obtained from biotopes of Svalbard waters with different temperatures and salinities; their results suggest that these lipids play a role in the compensatory mechanisms of adaptation.

A heterogeneity in the lipid spectra (particularly PL) dependent on the species and place of habitation was observed: slender eel blenny from both areas of the White Sea (Tersky shore and Onega Bay) were distinguished by a high level of SFM compared with the daubed shanny from Isfjord (Svalbard), and the amount of total PLs, including PI, PS, PEA, PC and LPC, was higher in slender eel blenny from Onega Bay than in slender eel blenny from the Tersky shore (Table 1). The detected differences in the lipid status of slender eel blenny from the White Sea was mainly dependent on the different trophoecological and hydrobiological features of the White Sea and were associated with biomembrane modifications that were caused by an increase or decrease of the physiological functions of the fishes. The differences between the two fish species suggested the existence of genetically determined processes of biosynthesis and modifications of some PLs (SFM).

Fatty acids are the most vital and sensitive components of lipids that actively participate in the development of the compensatory reactions of organisms in different ecological conditions. The investigation of the functional role of the fatty acids of lipids in the adaptation mechanisms of fishes is currently one of the most important aspects of physiological and biochemical research. It is known that the main determinant of the fatty acid (FA) spectra of fish lipids is the FA content of the available food at the low trophic levels [28,29].

The monogenic FAs (MUFAs) of total lipids dominate the FA classes in the daubed shanny from Svalbard waters and the slender eel blenny from the White Sea (from 38.8% to 42.7% of the total FAs) (Table 2). The prevalence of MUFA is a specific feature of the FA content of marine organisms that live at high latitudes and low temperatures. In addition, the 18:1(n-9) and 16:1(n-7) MUFAs were the most abundant FAs, and no significant differences (*p* ≤ 0.05) were detected in the concentrations of these MUFAs among the studied fishes. The 18:1(n-7) FA accounted for 5.7% to 7.7% of the total FAs and was significantly (*p* ≤ 0.05) more abundant in the sub-Arctic *Lumpenus fabricii* from the Tersky shore compared with the fishes from Onega Bay of the White Sea and the Arctic *Leptoclinus maculatus* from Isfjord (Table 2). These FAs are biomarkers for various species of benthic organisms that derive their FAs from bacteria and phytoplankton [30]. Certain benthic organisms serve as food for bottom-dwelling daubed shanny. It is known that bacteria, as one of the biotic components of benthic ecosystems, are considered a main source of monogenic 18:1(n-9) and 18:1(n-7) FAs, which are products of the desaturation of 18:0 and the elongation of 16:1(n-7), respectively [31,32]. Some MUFAs are biomarkers for specific groups of hydrobionts; for example, 18:1(n-9) is a biomarker of dinoflagellates and bacterioplankton and 16:1(n-7) is a biomarker of diatom microalgae [33–35]. Through trophic interactions, these MUFAs from bacteria and phytoplankton are transferred to decapods and polychaetes [30] and the latter serve as a fundamental food source for bottom-dwelling daubed shanny (own observations). The presence of a phytoplankton FA biomarker in Arctic daubed shanny observed in this study could be explained by the physiology and behavior of other fishes in the Stichaeidae family, which exhibit vertical migrations during their lifecycle [5]. The sub-Arctic slender eel blenny from the White Sea was collected at relatively shallow depths (38 m), where phytoplankton is accessible for feeding. The range of observed 18:1(n-9)/18:1(n-7) ratios suggests that the Arctic *Leptoclinus maculatus* and the sub-Arctic *Lumpenus fabricii* preferentially feed on zooplankton or animals low in the food chain. These fishes exhibited a highly diversified diet in the different investigated areas in autumn; this range is also indicative of the lipid content of the diet and makes it difficult to identify the food sources of the fishes. Some authors have noted the ineffectiveness and complexity of using trophic biomarkers in research of the FA status of higher-trophic-level predators, because the manner by which predators obtain certain trophic biomarkers and the direct *vs*. indirect nature of certain food webs remain unclear [36]. The same problem most likely exists in the analysis of fishes from the Stichaeidae family. This problem can only be resolved by a detailed investigation of the food sources of these fishes and the analysis of the movement and transformation of the lipids and their components through trophic webs of the marine organism in the sub-Arctic and high-Arctic.

Many authors have reported that 18:1 FAs are the most important lipids for cold-water organisms with respect to their adaptation to temperature and depth [37–39]. Lapin and Shatunovskii [37] emphasized the relative abundance of 18:1 FAs and the MUFA/polyenic FA (PUFA) ratio in the total FA content of lipids (especially TAG and wax esters) in fishes in deep habitats. We observed that the MUFA/PUFA ratio was higher in Arctic *Leptoclinus maculatus* that dwelled at temperatures of 0 °C and at depths of 206.0 m than in sub-Arctic *Lumpenus fabricii* from the White Sea (temperature of 5.9 to 6.7 °C and depth of 38.0 m).

It is important to note that 20:1(n-9) and 22:1(n-11), which are *Calanus* zooplankton biomarkers, comprised a minor component at 0.60% to 2.82% of the total FAs (Table 2). These FAs are synthesized *de novo* by Calanus copepods (*Calanus glacialis* and *Calanus finmarchicus*) and fish obtain these FAs by feeding [35,40]. We previously demonstrated that the larvae of *Leptoclinus maculatus* from Svalbard waters have a relatively high content of 20:1(n-9) and 22:1(n-11) at 28% and 20%, respectively, which reflects the zooplankton diet of this fish [27]. When the pelagic larvae of the fish develop to become bottom-dwelling adults, their food preferences changes. The food sources of the bottom-dwelling adult *Leptoclinus maculatus* and *Lumpenus fabricii* that inhabit marine ecosystems of high latitudes are not yet clearly understood. We hypothesize that the level of MUFAs in the adult specimens of daubed shanny and slender eel blenny studied herein are also influenced by other abiotic and biotic factors, such as the photoperiod, oxygen regime, salinity and availability of food resources.

The importance of lipids in the adaptation of fishes to a changing environment is indicated by the optimal values of the FA ratios. These ratios mainly depend on the FA content of food and the ability of the organism to modify its FAs in response to external and internal conditions. The saturated FA (SFA)/unsaturated FA (UFA) ratio plays an important role in determining various properties of biomembranes, such as the fluidity, that help maintain a normal metabolism in cells. In this research, the 16:0/18:1(n-9) ratios were 1.26, 1.37 and 1.18 for the daubed shanny from Isfjord, the slender eel blenny from Tersky shore and the slender eel blenny from Onega Bay, respectively (Table 2); these ratios reflect the level of activity of the lipid metabolism of these fishes [41].

Differences in the contents of PUFA and its metabolism were found between the two studied fish species. The Arctic *Leptoclinus maculatus* from Isfjord had a low amount of PUFAs (26.2% of the total FA) with a low level of (n-6) FAs (metabolically connected 18:2(n-6) and 20:4(n-6)) compared with the sub-Arctic *Lumpenus fabricii* from the White Sea (33.9% to 34.7% of the total FAs) (Table 2). One of the main reasons for these differences may be the different FA concentrations and ratios of their food. Among other ecological factors, the qualitative content of food sources has been emphasized as a principal factor that defines the PUFA level in the tissues of fishes [4,42]. The two-fold higher 18:2(n-6) content and the similar level of linoleic FA metabolite (20:4(n-6)) in the high-Arctic daubed shanny compared with the sub-Arctic slender eel blenny indicates the lower activity of linoleoyl-CoA desaturase, which plays a key role in the turnover of 18:2(n-6) to 20:4(n-6) [43]. Thus, the 20:4(n-6)/18:2(n-6) ratio, which indicates the activity of linoleoyl-CoA desaturase, was higher in the Arctic daubed shanny (3.76) than in the sub-Arctic slender eel blenny from the Tersky shore (3.0) and Onega Bay (2.7). The investigated fish species from the Stichaeidae family, which were collected from different areas, exhibited no significant differences in their (n-3) PUFA contents; however, the amount of 22:5(n-3) was higher (*p* ≤ 0.05) in the Arctic *Leptoclinus maculatus.*

The saturated FAs in the two studied species accounted for 25.0% to 27.3% of the total FAs. The 16:0 level was representative of the SFAs (15.0% to 15.7% of the total FAs) and no significant difference in the concentration of this FA among the fishes was observed (Table 2). The sub-Arctic *Lumpenus fabricii* differed (*p* ≤ 0.05) from the other studied fish due to the prevalence of 18:0.

## 3. Experimental Section

*Lumpenus fabricii* adults (females) were collected in October from two areas of the White Sea: Onega Bay (64°59′ 36°37′) and the Tersky Shore (66°05′ 37°38′). *Leptoclinus maculatus* adults (females) were obtained in October from Isfjord, Svalbard (78°39′ 16°37′) using a bottom trawl. Some characteristics of the sampling areas presented in Table 3.

The flesh of the fish was homogenized in 10 volumes (10 mL each) of 96% ethyl alcohol mixed with 0.001% of the antioxidant (ionol). The homogenates were placed in glass vials and stored onboard the ship in a cold room at 4 °C until delivery to the laboratory. The material was then fixed in a solvent of chloroform:methanol (2:1, *v*/*v*) and the total lipids (TLs) were extracted using the method [44]. The residues recovered after the lipid extraction of the tissues were dried over phosphoric anhydride until the samples reached a constant weight. The residues were weighed (*X*1) to determine the approximate percentage of total lipid on a dry-weight basis:

(1) Total lipids (% dry-weight)=X2×100/(X1+X2)

where *X*1 = residue weight (g); *X*2 = lipid extracted (g).

The lipid status of each fish was evaluated by the determination of the content of total lipids, triacylglycerides (TAGs), phospholipids (PLs), including the separate phospholipid classes (phosphatidylcholine (PC), phosphatidylethanolamine (PEA), phosphatidylserine (PS), phosphatidylinositol (PI), lysophosphatidylcholine (LPC) and sphingomyelin (SFM)) and the fatty acid spectrum.

Thin-layer chromatography was used to identify the lipid classes as PLs, TAGs, cholesterols (CHs) and wax esters combined with cholesterol esters (WE + CE). After drying, the chromatogram is developed in iodine vapor, which stains lipids yellow. These molecules were quantified using the hydroxamate method that was modified by [45], which involves the formation of dark-brown complexes of trivalent iron ions with hydroxamic acid through ester bonding between the lipids and hydroxylamine [46]. The stain intensity was measured using a spectrophotometer (SF-2000) at a wave length of 540 nm. The quantitative determination of CHs was determined based on the method described by [47] using trichloroacetic iron dissolved in perchloric acid. The stain intensity was measured using a spectrophotometer at a wavelength of 550 nm. Standards (Sigma Aldrich, St. Louis, MO, USA) for thin-layer chromatography were used to distinguish the lipid classes on the plates.

The chromatograms of individual phospholipid fractions were determined by high-performance liquid chromatography (HPLC) according to the method of [48] using a Nucleosil 100-7 column with a acetonitrile:hexane:methanol:phosphorus acid (918:30:30:17.5 by volume) mobile phase. The detection was performed using a spectrophotometer (UV light, 206 nm). Phospholipids standards (Supelco-Analytical, Belleponte, PA, USA) were used for the identification and quantification of the phospholipid compounds in the sample. We identified six phospholipids: phosphatidylserine, phosphatidylethanolamine, phosphatidylinositol, phosphatidylcholine, lysophosphatidylcholine and sphingomyelin.

The fatty acid composition of the total lipid extracts was analyzed by gas-liquid chromatography. Fatty acid methyl esters (FAME) were identified using a “Chromatec-Crystal-5000.1” (Shimadzu, Kyoto, Japan) gas chromatograph with a flame-ionization detector and a Zebron capillary gas chromatographic column (Phenomenex, Torrance, CA, USA). An isothermal column configuration was used (205 °C); the temperature of the detector and evaporator were 250 °C and 240 °C, respectively. The internal standard was 22:0 FA. Chromatec-Analytik-5000.1 software, version 2.6 (Yoshkar-Ola, Russia) was used for recording and integrating the data. FAME was identified with standard mixtures Supelco 37 FAME mix (Supelko-Analytical, Belleponte, PA, USA) and in the way of comparing of equivalent the length of carbon chain and table constants according Jamieson [49].

The research was carried out using the facilities of the Equipment Sharing Centre of the Institute of Biology, KarRC of RAS. The data were analyzed to determine whether they exhibited a normal distribution. The differences between the means of the lipid and fatty acid parameters of the fishes for the studied areas were analyzed by ANOVA (*p* ≤ 0.05).

## 4. Conclusions

Therefore, the different environments (high-Arctic and sub-Arctic) did not affect the level of total MUFAs, but influenced the level and degree of variation of the FAs 18:1(n-7), 20:1(n-7) and 22:1(n-11) in daubed shanny (*Leptoclinus maculatus*) and eel blenny (*Lumpenus fabricii*). The extent of saturation and the signature of polyenic FAs varied according to the specific species of the Stichaeidae family: the content of the total PUFAs, which was determined by the levels of (n-6) PUFAs [18:2(n-6) and 20:4(n-6)], was lower in the Arctic *Leptoclinus maculatus*, which exhibited a level of the minor 22:5(n-3) that was statistically higher.

The observed differences in the lipid spectra (PLs and FAs) for the two species from the Stichaeidae family may be associated with the unique characteristics of the genetically determined processes of biosynthesis and modifications of certain PLs and FAs, as well as the ecology (e.g., feeding and the oceanographic environment of habitats). These results demonstrate the importance of considering certain trends in the lipid profiles of these fishes as specific features of the organization of the ecological and biochemical mechanisms of adaptation to physiological limits.

## Figures and Tables

**Table 1:** Total lipids, triacylglycerols, wax esters, cholesterol esters, cholesterol, total phospholipids and separate phospholipids (% dry weight [dw]) in flesh of *Leptoclinus maculatus* from Svalbard (Isfjord) and *Lumpenus fabricii* from the White Sea (Onega Bay and Tersky Shore).

Parameter/Species, Area	*Leptoclinus maculatus*, svalbard (S)	*Lumpenus fabricii*, tersky shore of the white sea (T)	*Lumpenus fabricii*, onega bay of the white sea (O)
*n*	11	21	14
TL	11.6 ± 1.3	11.4 ± 0.5	11.7 ± 1.4
TAG	2.1 ± 0.6	5.8 ± 0.3 SO	2.5 ± 0.5
WE + CE	1.2 ± 0.3 TO	0.3 ± 0.0 SO	0.7 ± 0.1 ST
CH	3.6 ± 1.2 TO	0.9 ± 0.1 SO	1.7 ± 0.4 ST
PL	4.7 ± 0.9	4.4 ± 0.2 O	6.8 ± 0.9 T
PI	0.08 ± 0.0	0.03 ± 0.0 O	0.2 ± 0.0 T
PS	0.05 ± 0.0 O	0.04 ± 0.0 O	0.1 ± 0.0 TS
PEA	0.8 ± 0.2 T	0.4 ± 0.02 OS	0.7 ± 0.2 T
PC	3.5 ± 0.6 T	2.4 ± 0.1 OS	4.2 ± 0.7 T
LPC	0.05 ± 0.0 TO	0.9 ± 0.0 OS	1.3 ± 0.2 TS
SFM	0.03 ± 0.0 TO	0.2 ± 0.0 S	0.2 ± 0.0 S
Unknown	0.1 ± 0.0 TO	0.5 ± 0.1 OS	0.3 ± 0.0 TS

Data are means: *M* ± *m*; TAG: triacylglycerols; WE + CE: wax esters plus cholesterol esters; CH: cholesterol; PL: phospholipids; PC: phosphatidylcholine; PEA: phosphatidylethanolamine; PI: phosphatidylinositol; PS: phosphatidylserine; SFM: sphingomyelin; LPC: lysophosphatidylcholine. Values in the same row with the different letters are significantly different (*p* ≤ 0.05) among fishes from different areas:

S from fishes from Svalbard;

T from fishes from Tersky Shore of the White Sea;

O from fishes from Onega Bay of the White Sea.

**Table 2:** Fatty acid (FA) spectrum (% of total FA) in flesh of *Leptoclinus maculatus* from Svalbard (Isfjord) and *Lumpenus fabricii* from the White Sea (Onega Bay and Tersky Shore).

FA/species, areas	*Leptoclinus maculatus*, svalbard (S)	*Lumpenus fabricii*, tersky shore of the white sea (T)	*Lumpenus fabricii*, onega bay of the white sea (O)
14:0	2.28 ± 0.52 TO	2.95 ± 0.09 SO	3.77 ± 0.26 ST
16:0	15.71 ± 0.61	15.17 ± 0.21	15.00 ± 1.92
18:0	4.50 ± 0.19 TO	6.45 ± 0.24 SO	3.50 ± 0.16 ST
∑ SFA	25.35 ± 1.10 T	27.34 ± 0.39 S	24.98 ± 2.29
16:1(n-7)	11.02 ± 1.30	9.43 ± 0.32	9.37 ± 0.79
18:1(n-9)	12.45 ± 1.08	11.04 ± 0.43	12.69 ± 2.81
18:1(n-7)	6.32 ± 0.26 T	7.68 ± 0.19 SO	5.70 ± 0.87 T
20:1(n-9)	2.12 ± 0.71	2.02 ± 0.09	2.82 ± 1.15
20:1(n-7)	3.35 ± 0.67 T	2.10 ± 0.13 S	1.72 ± 0.53
22:1(n-11)	1.91 ± 1.23 T	0.60 ± 0.08 SO	1.43 ± 0.81 T
∑ MUFA	42.70 ± 4.09	38.77 ± 0.72	40.34 ± 1.90
18:2(n-6)	0.55 ± 0.02 TO	1.11 ± 0.04 S	1.06 ± 0.07 S
18:3(n-6)	0.41 ± 0.13	0.31 ± 0.02	0.31 ± 0.05
20:4(n-6)	2.07 ± 0.36 T	3.34 ± 0.15 S	2.83 ± 0.83
∑ (n-6)FA	4.65 ± 0.51 T	7.31 ± 0.18 S	6.38 ± 1.31
18:3(n-3)	0.42 ± 0.05	0.41 ± 0.02	0.55 ± 0.17
20:5(n-3)	13.94 ± 1.69 T	11.15 ± 0.37 S	11.53 ± 0.87
22:5(n-3)	2.79 ± 0.24 TO	1.63 ± 0.05 S	1.38 ± 0.29 S
22:6(n-3)	8.36 ± 1.31	8.11 ± 0.28	9.32 ± 0.91
∑ (n-3)FA	26.21 ± 3.01	23.62 ± 0.06	25.19 ± 0.54
∑ PUFA	26.21 ± 3.01 TO	33.89 ± 0.68 S	34.66 ± 0.82 S
∑ (n-6)/∑ (n-3)	0.18 ± 0.02 TO	0.31 ± 0.01 S	0.26 ± 0.05 S
18:3(n-3)/18:2(n-6)	0.77 ± 0.08 TO	0.36 ± 0.02 S	0.56 ± 0.22 S
16:0/18:1(n-9)	1.26 ± 0.16	1.37 ± 0.05	1.18 ± 0.09

Data are *M* ± *m*. Abbreviation: *n*: number of samples; FA: fatty acids; SFA: saturated fatty acids; MUFA: monounsaturated fatty acids; PUFA: polyunsaturated fatty acids. Values in the same row with the different letters are significantly different (*p* ≤ 0.05) among fishes from different areas:

S from fishes from Svalbard;

T from fishes from Tersky Shore of the White Sea;

O from fishes from Onega Bay of the White Sea.

**Table 3:** Some characteristics of the sampling areas.

Sampling area	Isfjord, svalbard	Tersky shore of the white sea	Onega bay of the white sea
Coordinates	78°39′ 16°37′	66°05′ 37°38′	64°59′ 36°37′
Depth, m	206	38	38
Temperature, °C	0	5.9	6.7
Salinity, ‰	35.0	27.0	26.4
